# MicroRNA-200c Promotes Suppressive Potential of Myeloid-Derived Suppressor Cells by Modulating PTEN and FOG2 Expression

**DOI:** 10.1371/journal.pone.0135867

**Published:** 2015-08-18

**Authors:** Shiyue Mei, Jiaxuan Xin, Yu Liu, Yuan Zhang, Xue Liang, Xiaomin Su, Hui Yan, Yugang Huang, Rongcun Yang

**Affiliations:** 1 Department of Immunology, Nankai University School of Medicine, Nankai University, Tianjin, P. R. China; 2 State Key Laboratory of Medicinal Chemical Biology, Nankai University, Tianjin, P. R. China; 3 Key Laboratory of Bioactive Materials Ministry of Education, Nankai University, Tianjin, P. R. China; Sun Yat-sen University Medical School, CHINA

## Abstract

Myeloid-derived suppressor cells (MDSCs) constitute one of the major populations that potently suppress anti-tumor immune responses and favor tumor growth in tumor microenvironment. However, the mechanism(s) regulating the differentiation and suppressive function of tumor-associated MDSCs remain(s) unclear. Here, we identified a microRNA-200c (miR-200c), whose expression was dramatically induced by tumor-derived factors. Meanwhile, we also demonstrated that GM-CSF was a main inducer of miR-200c in tumor environment, and miR-200c in turn promoted the expansion and immune suppressive activity of MDSCs via targeting phosphatase and tensin homolog (PTEN) and friend of Gata 2 (FOG2), which can lead to STAT3 and PI3K/Akt activation. Finally, we examined *in vivo* suppressive function of miR-200c transfected MDSCs and found that miR-200c could remarkably promote tumor growth via modifying MDSCs. Thus, GM-CSF induced miR-200c in tumor environment plays a critical role in governing the expansion and functions of tumor-associated MDSCs and serves as a potential target in immunotherapy against tumor.

## Introduction

Myeloid derived suppressor cells (MDSCs) accumulate in large numbers in tumor microenvironment and represent a key player mediating tumor immune tolerance [1–3]. In mouse, MDSCs are characterized by the simultaneously expression of the myeloid markers Gr-1 and CD11b [4], which are further subdivided into two subsets including mononuclear cells (MO-MDSCs) with a phenotype of inflammatory monocytes, expressing Ly6C marker (CD11b^+^Ly6G^-^Ly6C^high^) and polymorphonuclear cells (PMN-MDSCs) with a phenotype of immature neutrophils, expressing Ly6G marker (CD11b^+^Ly6G^+^Ly6C^low^) [3, 5]. In human, the phenotype of MDSCs are less well defined, but are generally regarded as CD11b^+^CD14^-^CD33^+^ cells or Lin^-^HLA^-^DR^-^CD33^+^ cells [6]. Two main subpopulations of MDSCs in patients have been recently also identified as CD14^+^ monocytes and CD15^+^ neutrophils MDSCs [7]. These MDSCs may suppress immune responses by producing reactive oxygen species (ROS), H_2_O_2_, NO- and arginase. Studies have shown that tumor associated factors such as GM-CSF, M-CSF, VEGF, stem cell factor (SCF), TNFα, IL-6, IFNγ, IL-1β, IL-4 and prostaglandin E2 (PGE2) can promote generation of MDSCs[8–12]. However, the mechanism controlling their differentiation and function in tumor environment is still not completely clear.

Two-signal models are proposed to be responsible for differentiation and suppressive function of MDSCs [13]. One signaling pathway is mainly for MDSC expansion and the other for MDSC activation. Tumor-derived cytokines, such as GM-CSF, M-CSF, G-CSF, IL-6 and VEGF, initiate the first kind of signaling pathways that converge on activation of STAT3 and STAT5. Because of the importance of these transcription factors on cell differentiation and proliferation, signals to them naturally prevent normal myeloid development and promote the expansion of immature myeloid cells. STAT3 regulates transcription of anti-apoptotic or pro-proliferative factors including BCL-XL, survivin, cyclin D1 and c-myc that are involved in myeloid cell expansion [1, 14]. Activation of STAT3 also upregulates pro-inflammatory proteins S100A8, S100A9 and NADPH oxidase (Nox2) complex, which play a role in the differentiation and suppressive function of MDSCs [15, 16]. Pro-inflammatory molecules such as IFNγ, IL-1β, IL-4, PGE2 and LPS which may induce activation of STAT1, STAT6, PI3K/Akt and NF-κB, are important factors constituting signals start MDSC activation. Notably, PI3K/Akt pathway is also critical for the generation of tumor-expanded MDSCs [17]. A significantly high level of phosphorylated Akt may be detected in tumor-expanded MDSCs and increased Akt activity results in subsequent activation of NF-κB and mTOR, two players in controlling MDSC function [18]. Meanwhile, activation of Akt prolongs the lifespan of MDSCs by regulating the intrinsic apoptotic/survival signal [18].

microRNAs (miRNAs) form a large family of small non-coding RNAs that have emerged as key post-transcriptional regulators of gene expression in mammals [19]. So far, more than 100 different miRNAs have been found to be expressed in immune cells; they are directly implicated in molecular pathways involved in immune response in tumor environment [20]. Indeed, our previous studies [21–23] have found some miRNAs such as miR-223, miR-22, miR-503, miR-17-5p and miR-20a could regulate intracellular signal pathways to affect the differentiation and function of MDSCs. Here we show that miR-200c, whose genomic locus is located in fragile regions within two chromosomal clusters, including miR-200c-141 cluster and miR-200b-200a-429 cluster, strongly promotes the suppressive potential of MDSCs via targeting PTEN and FOG2, leading to subsequent STAT3 and PI3K/Akt activation. Meanwhile, we also show that GM-CSF is a main factor in inducing miR-200c expression in tumor environment. In the immune therapy against tumor, miR-200c inhibitor strongly reduces the suppressive potential of MDSCs and retards tumor growth. These results offer a new method for improving anti-tumor responses.

## Materials and Methods

### Mice

C57BL/6 and BALB/c mice (male, 4–8 weeks old) were obtained from Beijing Animal Center and maintained under specific pathogen-free conditions. Murine melanoma (B16), lung carcinoma (Lewis), ovarian carcinoma (1D8), colon carcinoma (CT26) and 4T1 breast tumor cell lines were obtained from American Type Culture Collection (ATCC). B16 melanoma, Lewis lung cancer, 1D8 ovarian cancer and CT-26 colon tumor were respectively established in C57BL/6 mice and in BALB/c mice by s.c inoculation of 5–10×10^5^ tumor cells. To generate tumor cell conditioned medium (TCCM), sub-confluent tumor cells were kept in medium with serum concentration (2%) for 48 h., and then TCCM was collected, aliquoted, and kept at -80°C until used. The animal experiments were performed in accordance with institutional guidelines and the study was approved by the ethics committee of Nankai University.

### Reagents

RPMI1640, DMEM, fetal bovine serum (FBS) and antibiotics were obtained from HyClone. Recombinant murine GM-CSF, IL-6, TNFα and IL-4 were purchased from Peprotech. PGE2 and 2-ME (2-Mercaptoethanol) were from Sigma. The following Abs were used, including PE-, or FITC- conjugated anti-Gr-1 (RB6-8C5), anti-CD11b (M1/70), anti-Ly6G (1A8), anti-Ly6C (AL21), anti-CD80 (16-10A1), anti-CD86 (GL1), anti- CD40 (3/23), anti-CD11c (N418), anti- PD-1 (J43), anti- B7-H1 (MIH1), anti-CD4 (L3T4), anti-CD8a (Ly-2) and isotype control Abs, which were obtained from BD Biosciences. Purified anti-GM-CSF Ab (MP1-22E9) was purchased from Biolegend.

### Flow cytometric analysis

Single cell suspensions were stained with the indicated FITC- or PE- labeled antibodies at 4°C for 25 min in phosphate buffer saline (PBS) with 1% fetal calf serum (FCS). Cells were then washed twice, resuspended in PBS with 1% paraformadehyde and 1% FCS, and kept at 4°C prior to flow cytometric analysis. Cells were analyzed on a FACScan flow cytometer (BD Biosciences).

### In vitro generation of MDSCs

BM cells were obtained as described previously [24]. Briefly, BM cells were collected by removing the femurs of mice, and flushing out the marrow in RPMI1640 medium. The red blood cells were lysed with ammonium chloride buffer. For induction of MDSCs, 2 × 10^6^ BM cells were plated into 6-well plates in medium. The medium was supplemented with different cytokines combinations, including GM-CSF (40 ng/ml) alone, GM-CSF (40 ng/ml) with IL-6 (40 ng/ml), TNFα (40 ng/ml), IL-4 (40 ng/ml) and PEG2 (1.0 μg/ml) or 25% v/v TCCM in the presence of 20 μM 2-ME, 10% FCS, and 1% penicillin and streptomycin.

### MDSCs isolated from B16, CT-26 and Lewis tumor

CD11b+Gr-1+MDSCs were isolated by biotinylated CD11b and Gr-1 mAbs together with anti-biotin- coated magnetic microbeads according to the manufacturer’s protocol (Miltenyi Biotec.). Purity of cell populations reached more than 90%.

### RNA isolation and real-time quantitative PCR

Total RNA was isolated using Trizol Reagent (Invitrogen). Real-time quantitative RT-PCR (qRT-PCR) was performed using SYBR RT-PCR kits (Takara) and CFX96 Real-Time PCR Detection System (Bio-Rad). The relative expression level of gene and miRNAs was normalized to that of endogenous control GAPDH mRNA and U6 small RNA, respectively. Fold changes were calculated using the 2^–ΔΔCt^ method. PCR reactions were conducted in triplicate for each sample. The primers for detecting all the genes and miRNAs were described in Table A in S1 File.

### Transfection

The cells were cultured on 6-well plate and transfected in the following day. For miRNA mimics, inhibitors, siRNAs, and control oligonucleotides (oligoes, Guangzhou RiboBio), cells were transfected with the indicated oligoes (100 nM) using the Hiperfect Transfection Reagent (Qiagen) according to the manufacturers’ instructions.

### Luciferase reporter assay

3'-UTR reporter plasmids were constructed by cloning the PTEN 3'-UTR sequence (Fig A in S1 File) into downsteam of firefly luciferase cassette in pSiCHECK-2 vector (Promega). HEK293T Cells were cultured in a 24-well plate at a density of 1 × 10^5^ cells/well before transfection, and then cells were cotransfected with the WT-3'-UTR reporter plasmids, mutated-3'-UTR reporter plasmids, control plasmids (Promega) respectively, and the indicated miRNAs using Lipofectamine reagent (Invitrogen) according to the manufacturers’ recommendations. Renilla and firefly luciferase activities were measured 24 h after transfection using the Dual-Luciferase Reporter Assay System (Promega). Renilla luciferase was normalized according to firefly luciferase activity.

### Arginase activity assay

Arginase activity was measured in cell lysates according to the reported protocol. Briefly, cells were lysed for 30 min with 100 μl of 0.1% Triton X-100 at 4°C. Following lysis, 100 μl of 25 mM Tris-HCl and 10 μl of 10 mM MnCl_2_ were added and the mixture was heated for 10 min at 56°C. Subsequently, the lysates were incubated with 100 μl of 0.5M L-arginine (pH 9.7) at 37°C for 120 min. The reaction was stopped with 900 μl of H_2_SO_4_ (96%)/H3PO4 (85%)/H_2_O (1:3:7). Urea concentration was measured by absorbance at 540 nm after addition of 40 μl of 9% α-isonitrosopropiophenone, followed by heating at 95°C for 30 min. A standard curve was generated by serial dilution of 120 mg/ml urea. Arginase activity (Unit) was defined by the amount enzyme that catalyzes the formation of 1ug of urea per minute.

### Nitric oxide production

Nitric oxide in culture supernatant was assayed using Griess Reagent System. Equal volumes of supernatant (50 μl) and sulfanilamide solution (1% sulfanilamide in 5% phosphoric acid) were incubated at room temperature (RT) for 5–10 min, followed by addition of 50 ul of NED solution (0.1% N-1-napthylethylenediamine dihydrochloride). After incubation for 10 min at RT, absorbance at 550 nm was measured. Nitrite concentrations were quantified by comparing the absorbance values to a standard curve generated by serial dilution of 100 uM sodium nitrite.

### H_2_O_2_ production

Production of H_2_O_2_ was evaluated using Amplex Red Hydrogen Peroxide/ Peroxidase Assay Kit (Invitrogen). Briefly, 1×10^4^ cells were resuspended in Hanks Balanced Salt Solution. After addition of PMA (30 ng/ml), the absorbance at 560 nm was measured using a microplate reader at 37°C. Absorbance values for the test samples were normalized to a standard curve generated by serial dilutions of 10 mM H_2_O_2_.

### ROS detection

Oxidation-sensitive dye DCFDA was used to measure ROS production by MDSCs. Cells were incubated at 37°C in RPMI medium in the presence of 2.5 μM DCFDA for 30 min. For phorbol myristate acetate (PMA)-induced activation, cells were simultaneously cultured with DCFDA and 30 ng/ml PMA and then flow cytometric analysis was then performed.

### Western blotting

Cell lysates were denatured and subjected to SDS-PAGE, and then were transferred to PVDF membranes. The membranes were incubated with the primary Abs, followed by hybridization with the secondary HRP-conjugated Abs. Detection was performed using an enhanced chemiluminescence assay with Lumi-Glo reagents (Millipore). Anti-ARG1,-iNOS,-PTEN and-FOG2 antibodies were obtained from GeneTex. Anti-S100A8 and-S100A9 antibodies were from Abcam. Antibodies for STAT3, Akt, NF-κB P65, β-actin and phosphorylated STAT3, Akt, NF-κB p65 were purchased from Santa Cruz Biotechnology.

### Proliferation assay

CD4^+^ or CD8^+^ T cells were isolated from splenocytes by biotinylated CD4 and CD8 mAbs together with anti-biotin- coated magnetic microbeads by using Miltenyi Biotec kits and LS columns, and then were resuspended in pre-warmed (37°C) PBS/0.1% FCS containing CFSE (5 uM), and incubated 5 min at 37°C. The reaction was quenched with ice-cold culture media and incubated 10 min on ice. The cells were washed three times and harvested for further analysis.

For T cell suppression assay, CFDA-SE labeled CD4 ^+^ or CD8^+^ T cells from splenocytes were seeded in triplicates in 96-well round bottom plates pre-coating with anti-CD3ε antibody (BD Biosciences). CD4^+^ or CD8^+^ T cells (1×10^6^/well) were cultured in the presence of increasing MDSCs. CFDA-SE dilution in T cells was analyzed by flow cytometry after three days.

### In vivo MDSC suppressive experiments

Effects of tumor-associated miRNAs on suppressive potential of MDSC was assessed according to the previously reported protocol [25]. Mice bearing CT-26 tumors were randomly divided into several experimental groups (six mice per group) and treated via intratumoral injection with 2 × 10^6^ pretreated bone marrow derived MDSCs (BM-MDSCs) on days +5, +12, +19 after the inoculation of CT-26 tumor cells. MDSCs were transfected with miR-200c mimics, miR-200c inhibitors, FOG2 siRNAs (CTGGAGCAATAAAGAAGACAA) and PTEN siRNAs (GAAGTAAGGACCAGAGACA) or control oligoes (Guangzhou Ribobio, China). Tumor measurements were conducted by assessing the largest diameter and its perpendicular length with a caliper. The tumor size index was the average of these diameters. Mice were sacrificed on day 28.

### CD3- ζ Chain staining

For CD3- ζ Chain staining, T cell surface marker (CD4 and CD8) staining were performed prior to fixation and then cells were washed with PBS twice, followed by fixed with 1% paraformaldehyde in PBS for 15 min, permeabilized with 0.1% Triton X-100 for 10 min, and then washed in PBS. The fixed and permeabilized cells were incubated in 2% BSA/PBS for 30 min to block nonspecific binding. After washing, the cells were incubated with anti- CD3- ζ mAb for 1hr. All steps were performed at 4°C. Cells were analyzed on a FACScan flow cytometer.

### ELISA

ELISA for mouse GM-CSF was performed by using ELISA kit (Beijing 4A Biotech) following manufacturer’s instructions. The ODs of each samples were measured at 450nm using a SpectraMax 190 ELISA plate reader. GM-CSF levels were quantitated from three titrations using standard curves and expressed in picograms per milliliter.

### Statistical analysis

Student's t-test was used to determine significance. A 95% confidence interval was considered significant and was defined as P < 0.05. * p < 0.05, ** p < 0.01.

## Results

### miR-200c is highly expressed in tumor-associated MDSCs

**Fig 1:**
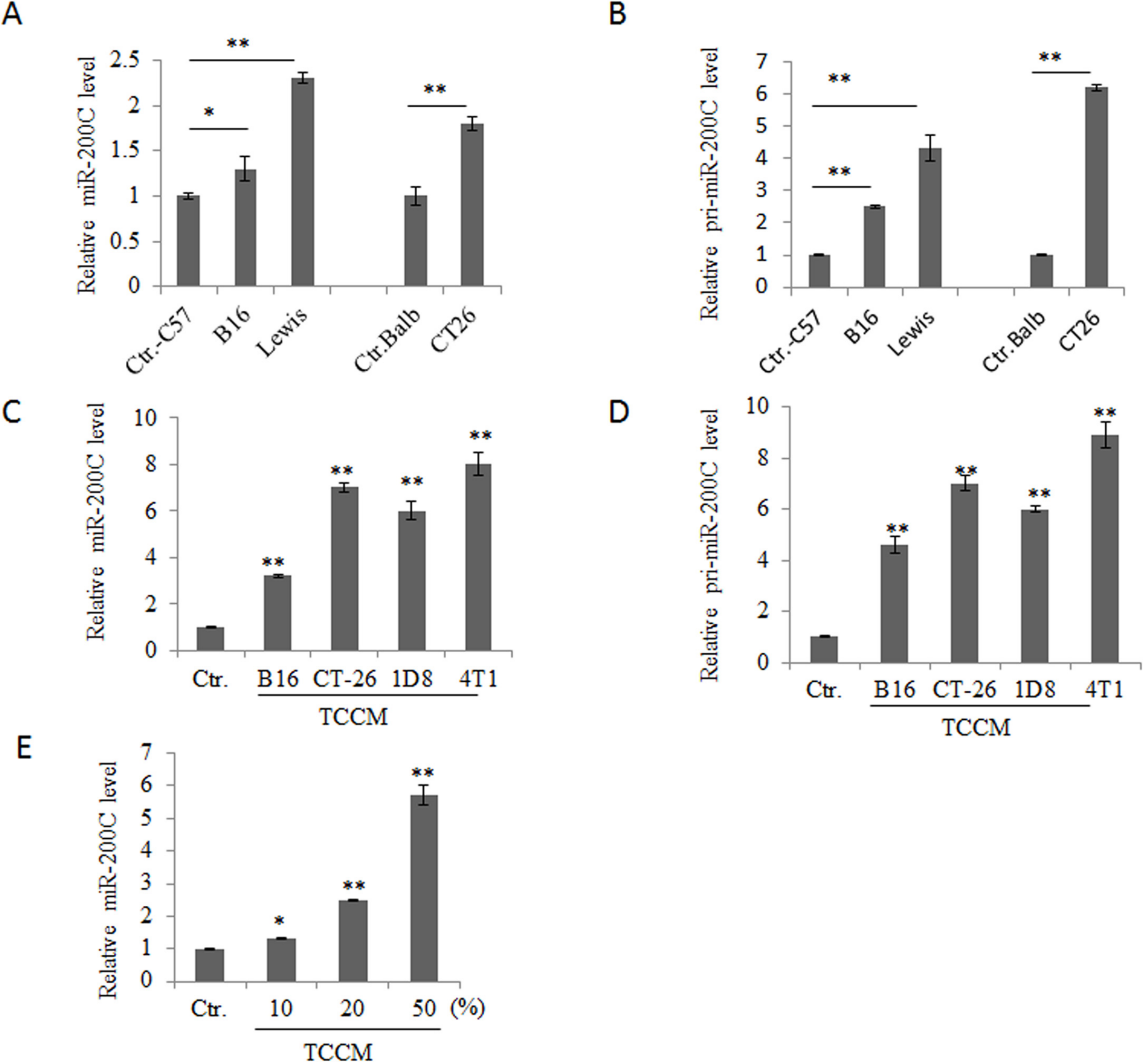
MiR-200c is highly expressed in tumor-associated MDSCs. (A and B) Expression of mature (A) and primary (B) miR-200c in CD11b^+^Gr-1^+^ cells isolated from the bone marrow of mice with different tumors. Ctrl-C57, B16 and Lewis were respectively CD11b^+^Gr1^+^ cells from tumor-free C57BL/6 mice, C57BL/6 mice bearing B16 melanoma and C57BL/6 bearing Lewis lung cancer; Ctrl-Balb andCT-26 were respectively CD11b^+^Gr1^+^ cells from tumor-free Balb/c mice and Balb/c mice with CT-26 tumor. (C and D) Expression of mature (C) and primary (D) miR-200c in tumor cell conditioned medium (TCCM) treated BM cells. BM cells were cultured with TCCM from B16 melanoma (B16), CT-26 colon cancer (CT-26), ID8 ovarian cancer (1D8) or 4T1 breast cancer (4T1, 25% v/v) for 36 h. (E) Expression of miR-200c in BM cells treated by different doses of TCCM. BM cells were treated with serial dilution of 4T1 TCCM for 36 h. 10, 20 and 50% indicated the concentrations of TCCM in medium. Each experiment was independently performed three times. *, p<0.05; **, P<0.01.

We selected microRNA-200c (miR-200c) as the focus microRNA (miRNA) of this study with the potential to regulate the differentiation and function of myeloid-derived suppressor cells (MDSCs) in tumor microenvironment because of the facts that miR-200c could be detected in myeloid derived CD11b^+^Gr-1^+^ cells cells [26]. We first investigated whether tumors could affect the expression of miR-200C in myeloid derived cells. To test this, we first established mouse tumor models, and analyzed the expression of miR-200C in the isolated CD11b^+^Gr-1^+^ cells from bone marrow (BM) cells of mice bearing these tumors. As shown in Fig 1A and 1B, increased expression of miR-200c could be detected in tumor-associated CD11b^+^Gr-1^+^ cells, which were isolated from bone marrow (BM) cells of mice bearing Lewis lung carcinoma, B16 melanoma or CT26 colon carcinoma. MiR-200c was also upregulated in BM cells after exposed to B16 melanoma, CT-26 colon carcinoma, ID8 ovarian cancer or 4T1 breast tumor cell conditioned medium (TCCM, 25% v/v), especially to that of CT-26 and 4T1 (Fig 1C and 1D). Furthermore, TCCM induced miR-200c expression was dose-dependent (Fig 1E). Thus, tumor-associated factors contributed to miR-200c upregulation, suggesting that miR-200c may be involved in the differentiation and suppressive function of tumor associated MDSCs.

### GM-CSF is a main inducer of miR-200c in tumor associated MDSCs

**Fig 2:**
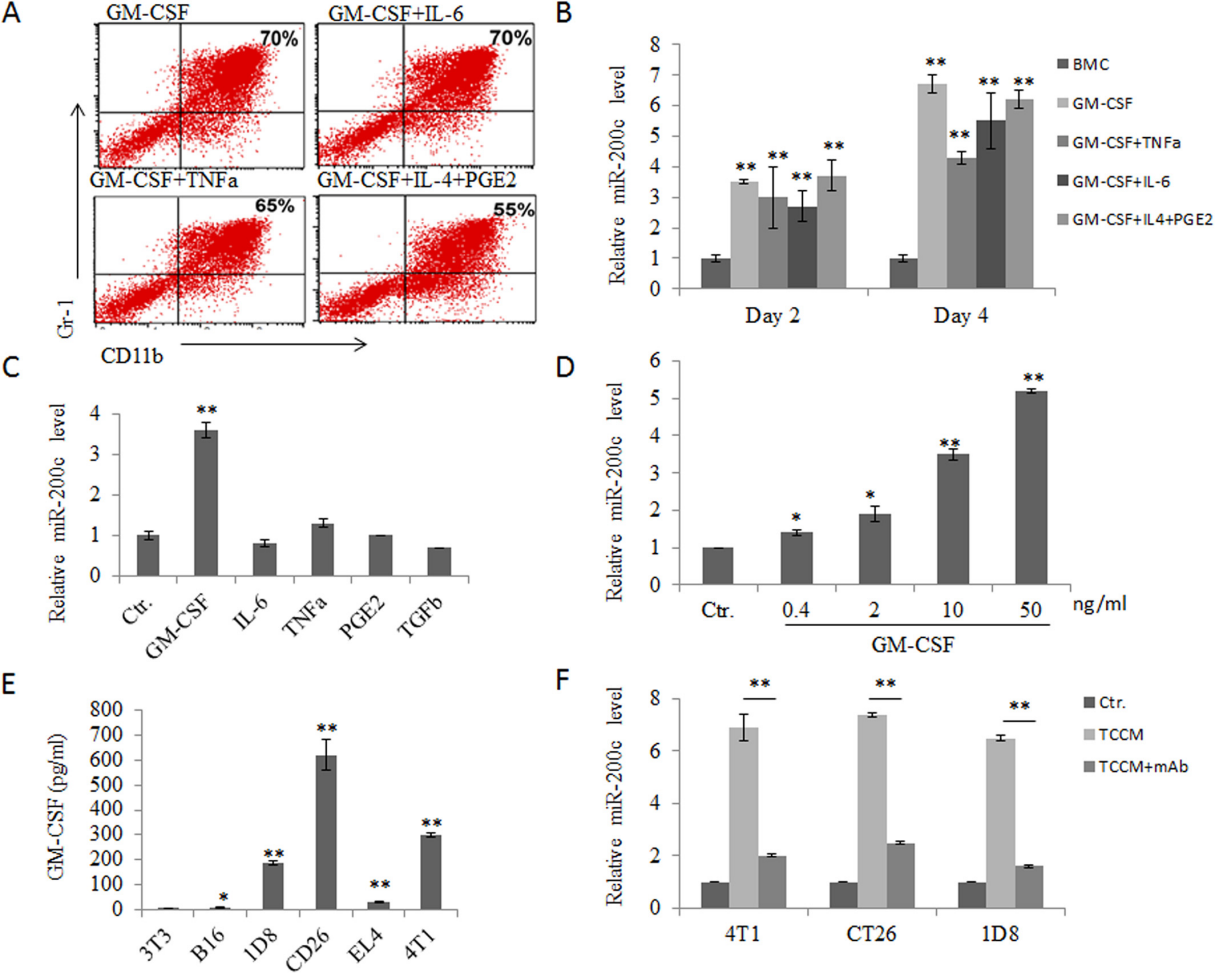
MiR-200c is upregulated by tumor associated GM-CSF. (A) Generation of BM cells derived CD11b^+^Gr1^+^ cells. BM cells derived CD11b^+^Gr1^+^ cells were generated by culturing with GM-CSF (40 ng/ml) or GM-CSF (40 ng/ml) with IL-6 (40 ng/ml), TNFα (40 ng/ml), or IL-4 (40 ng/ml) plus PGE2 (1.0 μg/ml). The proportion of Gr-1^+^CD11b^+^ cells was analyzed by FAScan. (B) Expression of miR-200c in differently derived MDSCs. BM cells derived CD11b^+^Gr1^+^ cells were generated by culturing with GM-CSF or GM-CSF with IL-6, TNFα, or IL-4 plus PGE2. Total RNAs were isolated from either fresh (fresh) or MDSCs (days 2 or 4 after exposed to the simulators) and miR-200c content was detected by qRT-PCR. Data are normalized to miRNA level in fresh BM cells. (C) Expression of miR-200c in different cytokines treated BM cells. BM cells were treated with different tumor-derived factors GM-CSF, IL-6, TNFα, PGE2 or TGFβ. Ctr., no tumor-derived factor. MiR-200c expression was examined after 24 h. (D) Expression of miR-200c in different doses of GM-CSF treated MDSC. BM cells were cultured with different concentrations of GM-CSF and then expression of miR-200c was analyzed. (E) GM-CSF levels in the supernatants of different tumor cell lines. The tumor supernatants were collected and analyzed by ELISA. (F) Effects of anti-GM-CSF on the expression of miR-200c. BM cells were cultured with 4T1, CT-26 or ID8 TCCM (25% v/v), without or with 10 μg/ml anti-GM-CSF Ab for 36 h. MiR-200c transcriptional levels were detected by qRT-PCR. Ctr., only medium; TCCM, medium with 25% tumor supernatants; TCCM+mAb, medium with 25% tumor supernatants plus anti-murine GM-CSF antibodies. Data are a representative of three independent experiments. *, p<0.05; **, P<0.01.

The data from both *in vivo* tumor models and *in vitro* induction suggest that tumor-associated factors contribute to miR-200c upregulation in MDSCs. However, it was still unclear which kind(s) of factor(s) in tumor environment is an inducer of miR-200c. To identify this, we first analyzed the expression of miR-200c in *in vitro* generated MDSCs from different combinations of tumor-derived factors including GM-CSF alone, GM-CSF with IL-6, GM-CSF with TNFα and GM-CSF with IL-4 plus PGE2 according to the protocols previously described [16, 27, 28] (Fig 2A). We found that miR-200c expression was higher in all MDSCs from different combinations of cytokines than that in untreated BMCs. As shown in Fig 2B, 2–3 fold high levels of miR-200c could be detected after culturing for 2 days. Continuing culture could continuously increase the expression of miR-200c to reach 5–6 folds as compared to control group. However, addition of TNFα, IL-6 or IL-4 and PEG2 was not able to promote the expression of miR-200c as compared to GM-CSF alone. In contrast, these factors reduced the expression of GM-CSF-mediated miR-200c expression in some degrees (Fig 2B), indicating that GM-CSF might be a main factor for the expression of miR-200c in tumor environment. To prove this, we investigated the effect of each tumor-derived factor on miR-200c expression respectively. Indeed, while cells were treated with IL-6, TNFα, TGFβ or PGE2, miR-200c expression did not show any significant change (Fig 2C). However, significant upregulation of miR-200c could be detected in GM-CSF-mediated MDSCs, and further this upregulation was dose-dependent (Fig 2D). Importantly, CT-26, 4T1 and 1D8 tumor cells could produce GM-CSF (Fig 2E), consistent with other findings[29, 30]. Furthermore, addition of anti-GM-CSF mAb into culture also partly inhibited the expression of miR-200c (Fig 2F). Taken together, these results suggest that the tumor-derived factor GM-CSF contributes to the upregulation of MDSC miR-200c in tumor environment.

### miR-200c enhances immune suppressive activity and differentiation of MDSCs

**Fig 3:**
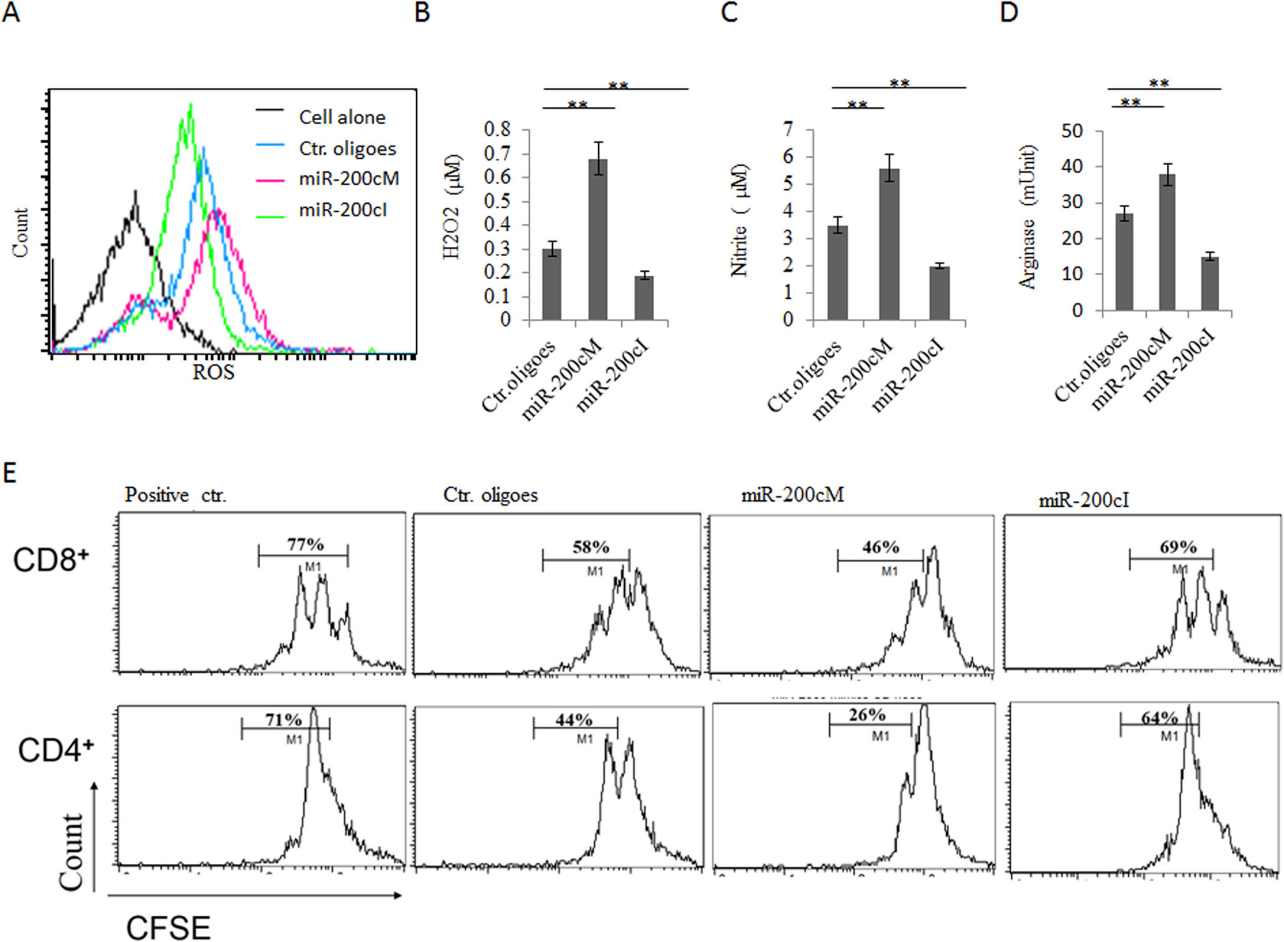
MiR-200c enhances immune suppressive function of MDSCs. (A, B, C and D) Production of ROS (A), H_2_O_2_ (B), NO- (C) and arginase activity (D) in differently transfected cells. BM cells were transfected with miR-200c mimics (miR-200cM), miR-200c inhibitors (Anti-200cI), or control oligoes (Ctr. oligoes) and then further cultured in the presence of GM-CSF and IL-6 for 4 days. ROS, H_2_O_2_, NO- and arginase activity were analyzed as described in Materials and Methods. (E) Suppressive function of differently treated MDSCs. CFDA-SE-labeled CD4^+^ or CD8^+^T cells were stimulated with anti-CD3 mAbs in the presence of MDSCs, which were pre-treated with either miR-200c mimics (miR-200cM), anti-200c inhibitors (miR-200cI) or control oligoes (Ctr. ologoes). Numbers % indicated the percentages of CD8^+^ or CD4^+^ proliferative cells. Positive Ctr., positive control without MDSCs. Data are a representative of three independent experiments. *, p<0.05; **, P<0.01.

Next, we addressed the effect of miR-200c on the differentiation and suppressive function of tumor associated MDSCs. It had been well-known that immunosuppression of tumor associated MDSCs was mainly related to the production of reactive oxygen species (ROS) including O_2_^-^, H_2_O_2_, ONOO^-^, nitric oxide (NO) and L-arginine metabolism[1]. Thus, we determined whether miR-200c could affect the generation of these immune suppressive substances. BM cells were transfected with miRNA mimics or inhibitors, and further cultured with GM-CSF and IL-6 for 3 days. The results showed that MDSCs pre-transfected with miR-200c mimics had an increased production of ROS, H_2_O_2_ and NO- (Fig 3A–3C). Meanwhile, miR-200c also enhanced arginase activity in MDSCs (Fig 3D). In contrast, miR-200c inhibitors not only reduced the level of ROS, H_2_O_2_ and NO- but also decreased the arginase activity (Fig 3A–3D). These molecules play a critical role in the suppression of T cell activation and proliferation, such as that ROS causes the loss of the T cell receptor (TCR) CD3ζ-chain [28, 31, 32] and that arginase 1 affects CD3ζ chain expression and inhibits TCR-mediated T cell responses [33]. Thus, we further tested the effect of miR-200c mimics loaded MDSCs on T cell activation. CD4^+^ T or CD8^+^ T cells were sorted from spleen, labeled by CFSE and stimulated with anti-CD3ε antibody, and then cocultured with differently modified MDSCs. As shown in Fig 3E, miR-200c mimics treated MDSCs showed remarkable suppression on T cell proliferation with a decrease in the percentage of proliferating cells; whereas miR-200c inhibitors alleviated the suppressive function of MDSCs (Fig 3E).

In addition, miR-200c also had an effect on the generation of MDSCs. As shown in Fig B in S1 File, while miR-200c mimics or control oligoes transfected BMCs were cultured for 3 days with different combinations of cytokines. miR-200c increased 10% of populations of CD11b^+^Gr-1^+^ cells as compared to control groups in the four induction models (GM-CSF with IL-6, TNFα, or IL-4 plus PGE2). Based on the fact that in normal condition, the population of CD11b^+^ Gr-1^+^ cells accounted for about 30% in fresh BM cells [28], 10% increment in CD11b^+^Gr1^+^ cells in miR-200c transfected group could be seen as significant. Notably, BMCs cultured in medium with only GM-CSF showed the most increment (>20%).

### miR-200c targets FOG2 and PTEN to affect the suppressive function and differentiation of MDSCs

**Fig 4:**
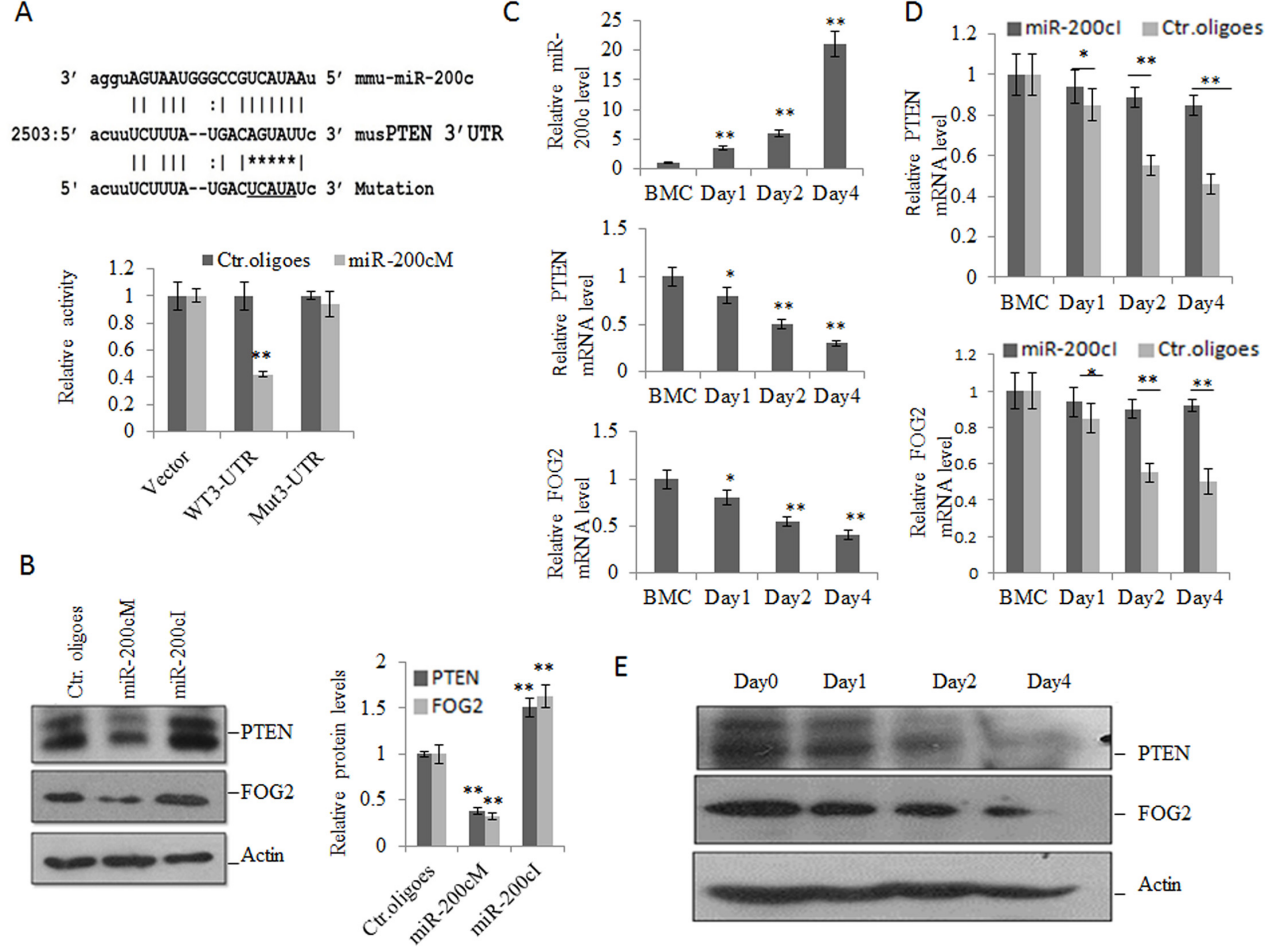
MiR-200c regulates the expression of FOG2 and PTEN. (A) Effect of miR-200c on the luciferase activity. 3’-UTR conserved site of PTEN and its mutated version for miR-200c pairing were exhibited in upper part. 293T cells were cotransfected with the luciferase reporter vectors and miR-200c mimics (miR-200cM) or control oligoes (Ctr.oligoes). The luciferase activity was measured after transfection for 36 hrs according to the protocol described in Materials and Methods (lower). Vector, pSiCHECK-2 vector; WT 3’-UTR, wild type PTEN 3’UTR; Mut-3’-UTR, PTEN 3’UTR with mutation in the putative miR-200c binding sites. (B) Effects of MiR-200c on the expression of PTEN and FOG2. MDSCs were transfected with miR-200c mimics (miR-200cM), miR-200c inhibitors (miR-200cI) or control oligoes (Ctr. oligoes),and then protein of PTEN and FOG2were detected by Western blot. The right histogram represents the relative densitometric value corresponding bands in the Western blot. (C) Effects of TCCM on the expression of miR-200c, PTEN and FOG2. BM cells were exposed to TCCM and then the expression of miR-200c (upper) and PTEN (middle) and FOG2 (lower) was detected at the indicated time points. BMC, bone marrow cells which were not exposed to TCCM. (D) Effect of anti-miR-200c on the expression of PTEN and FOG2 in TCCM treated BM cells. BM cells were transfected by miR-200c inhibitors (miR-200cI) or control oligoes (Ctr. oligoes), and then exposed to TCCM. Transcriptional levels of PTEN (upper) and FOG2 (lower) were detected by qRT-PCR at the indicated time. (E) Protein levels of PTEN and FOG2 in BM cells after exposed to TCCM. Protein levels of PTEN and FOG2 in BM cells after exposed to TCCM were detected by Western blot at the indicated time. Each experiment was independently performed three times. *, p<0.05; **, P<0.01.

We next determined the mechanism of miR-200c regulating the suppressive function and differentiation of MDSCs. Using TargetScan (http://www.targetscan.org/) prediction program combined with Pictar (http://pictar.mdc-berlin.de/), we identified 2 potential targets of miR-200c related to myeloid differentiation, FOG2 and PTEN. Both of them contained sequence motifs matching the “seed” sequence of miR-200c in their 3’ untranslational regions (3’UTRs) (Fig 4A and Fig C in S1 File) and were regarded as the most important negative regulators of PI3K/Akt signaling pathways, which were strictly related to the differentiation and suppressive function of MDSCs [25, 34, 35]. To test whether miR-200c was able to regulate PTEN directly, we constructed reporter plasmids carrying the wild type PTEN 3’UTR (pPTEN-WT), and plasmids with mutation in the putative miR-200c binding sites (pPTEN-MUT). While the reporter plasmids and miR-200c mimics or the control oligoes were co-transfected to 293T cells, miR-200c mimics decreased the luciferase activity by around 30% (Fig 4A). Importantly, the mutation in the putative miR-200c binding sites abolished the repression by miR-200c, demonstrating that miR-200c could specifically target their binding sites in the 3’UTR of PTEN. The protein levels of PTEN were also significantly down-regulated by miR-200c mimics (Fig 4B). Supporting above finding, anti-miR-200c also upregulated expression of PTEN (Fig 4B). To further determine that PTEN may be acted as targets of miR-200c, we further investigated the expression of miR-200c and PTEN in BM cells after exposed to TCCM. We found that miR-200c and PTEN expression levels exhibited a negative correlation. While miR-200c expression was increased gradually (Fig 4C), PTEN expression was decreased progressively at both mRNA and protein levels (Fig 4C and 4E). Meanwhile, we also found that miR-200c down-regulated the expression of FOG2 in both transcriptional and protein levels (Fig 4B, 4C and 4E). Importantly, miR-200c inhibitors abolished the suppression of miR-200C on PTEN and FOG2 in the presence of TCCM (Fig 4D). Taken together, these data demonstrate that miR-200c targets and reduces the levels of PTEN and FOG2 in MDSCs.

**Fig 5:**
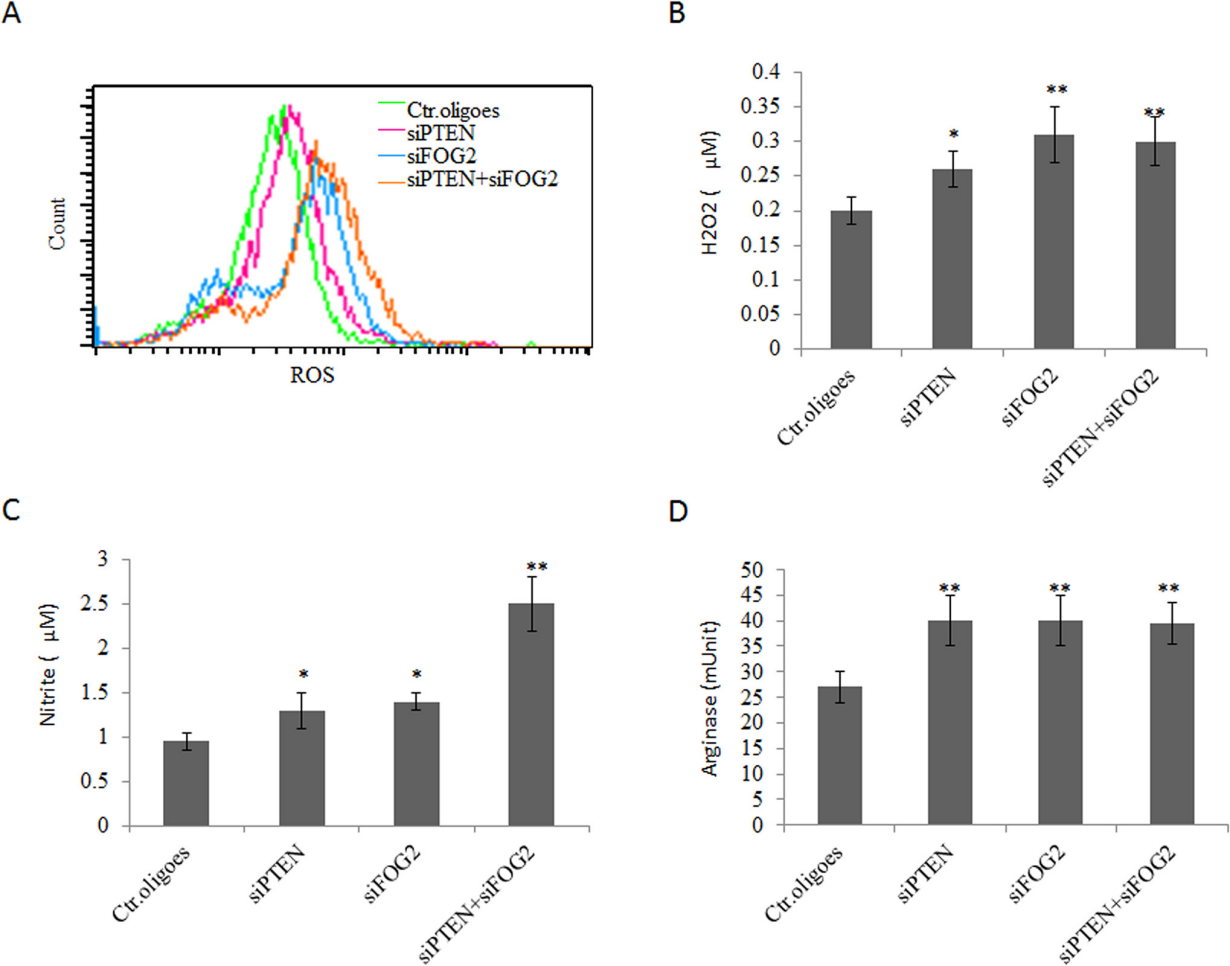
Silencing PTEN and FOG2 enhances immune suppressive activity of MDSCs. (A, B, C and D) Production of ROS (A), H_2_O_2_ (B), NO- (C) and arginase activity (D) in differently transfected cells. BM cells were transfected with FOG2 siRNA (siFOG2), PTEN siRNA (siPTEN) or both siFOG2 and siPTEN, and then cultured in the presence of GM-CSF and IL-6. The production of ROS, H_2_O_2_, NO- and arginase activity were measured. Ctr.oligoes,, control oligoes. The figures shown here are representative results from three independent experiments. *, p<0.05; **, P<0.01.

We next addressed whether miR200c mediated suppressive function in MDSCs was caused from PTEN and FOG2. Indeed, silencing PTEN or FOG2 using PTEN or FOG2 specific siRNAs could increase production of ROS, H_2_O_2_ and NO (Fig 5A–5C) and meanwhile, also enhanced the arginase activity (Fig 5D). Thus miR-200c could regulate differentiation and suppressive function of MDSCs by targeting PTEN and FOG2.

### miR-200c is involved in activation of PI3K/Akt and STAT3 in MDSCs

**Fig 6:**
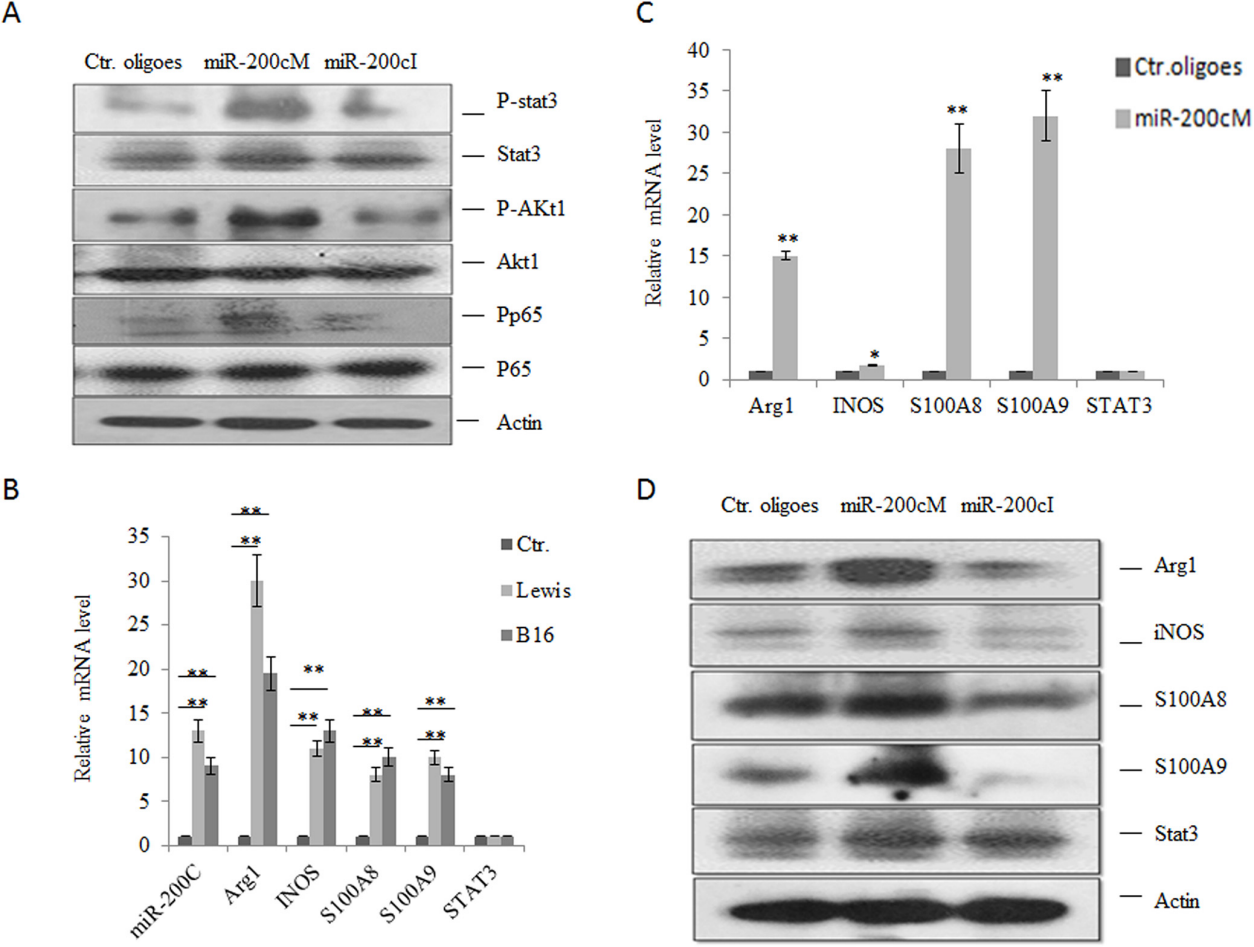
MiR-200c enhances the expression of immune suppression associated genes in MDSCs. (A) Phosphorylation of STAT3, Akt and p65 in differently transfected cells. BM cells were transfected with miR-200c mimics (miR-200cM), miR-200c inhibitor (miR-200c) or control oligoes (Ctr. oligoes) and then cultured in the presence of GM-CSF and IL-6. After further culturing for 3 days, phospho-STAT3,-Akt and-p65 were analyzed by Western blot. (B) Expression of immuno-suppression associated genes in CD11b^+^Gr-1^+^ cells isolated from spleens of mice with Lewis or B16 tumors. Ctr., CD11b^+^Gr-1^+^ cells isolated from spleens of tumor-free mice. (C and D) Expression of immune suppression associated genes in differently transfected BM cells. BM cells were transfected with miR-200c mimics (miR-200cM), miR-200c inhibitors (miR-200cI), or control oligoes (Ctr. oligoes) and then cultured with GM-CSF and IL-6. After further culturing for 3 days, immunosuppressive genes were detected by qRT-PCR (C) and Western blot (D). Each experiment was independently performed three times and for Western blotexperiments, the representative results are shown here. *, p<0.05; **, P<0.01

It has been well known that the NF-κB, STAT3 and PI3K/Akt signal pathways play a critical role in inducing the differentiation and suppressive function of MDSCs [1, 34]. Since both PTEN and FOG2 are regarded as important regulators of these signaling pathways [25, 34], we next analyzed the effects of miR-200c on the activity of Akt, NF-κB, and STAT3 in MDSCs. As shown in Fig 6A, transfection of miR-200c mimics dramatically enhanced the phosphorylation levels of Akt and STAT3; whereas miR-200c inhibitors reduced the activation of Akt and STAT3. Meanwhile, we also found that miR-200c could promote the phosphorylation of NF-κB subunit p65 (Fig 6A), consistent with the previous data that PTEN expression is inversely correlated with the phosphorylation levels of NF-κB[18].

Next, we analyzed the expression of MDSC associated genes such as ARG1, iNOS, S100A8 and S100A9, which may be regulated by Akt, NF-κB, and STAT3 signaling pathway [1, 34]. Tumor associated MDSCs were isolated from the spleens in Lewis lung carcinoma and B16 melanoma loaded mice. As shown in Fig 6B, higher levels of ARG1, iNOS, S100A8 and S100A9 in CD11b^+^Gr-1 ^+^ cells from Lewis- and B16- tumor bearing mice could be detected as compared to the counterparts from tumor-free mice. For *in vitro* GM-CSF and IL-6 induced MDSCs, miR-200c also dramatically increased the levels of ARG1, iNOS, S100A8 and S100A9 mRNAs (Fig 6C). Notably, there was not a significant change in the expression of STAT3, indicating that miR-200c was not involved in regulating its expression. These effects were further confirmed at the protein level (Fig 6D). Taken together, these results suggested that miR-200c regulated activation of Akt, NF-ΚB and STAT3 to control the expression of MDSC associated genes by targeting PTEN and FOG2.

### miR-200c promotes immune suppressive function of MDSCs

**Fig 7:**
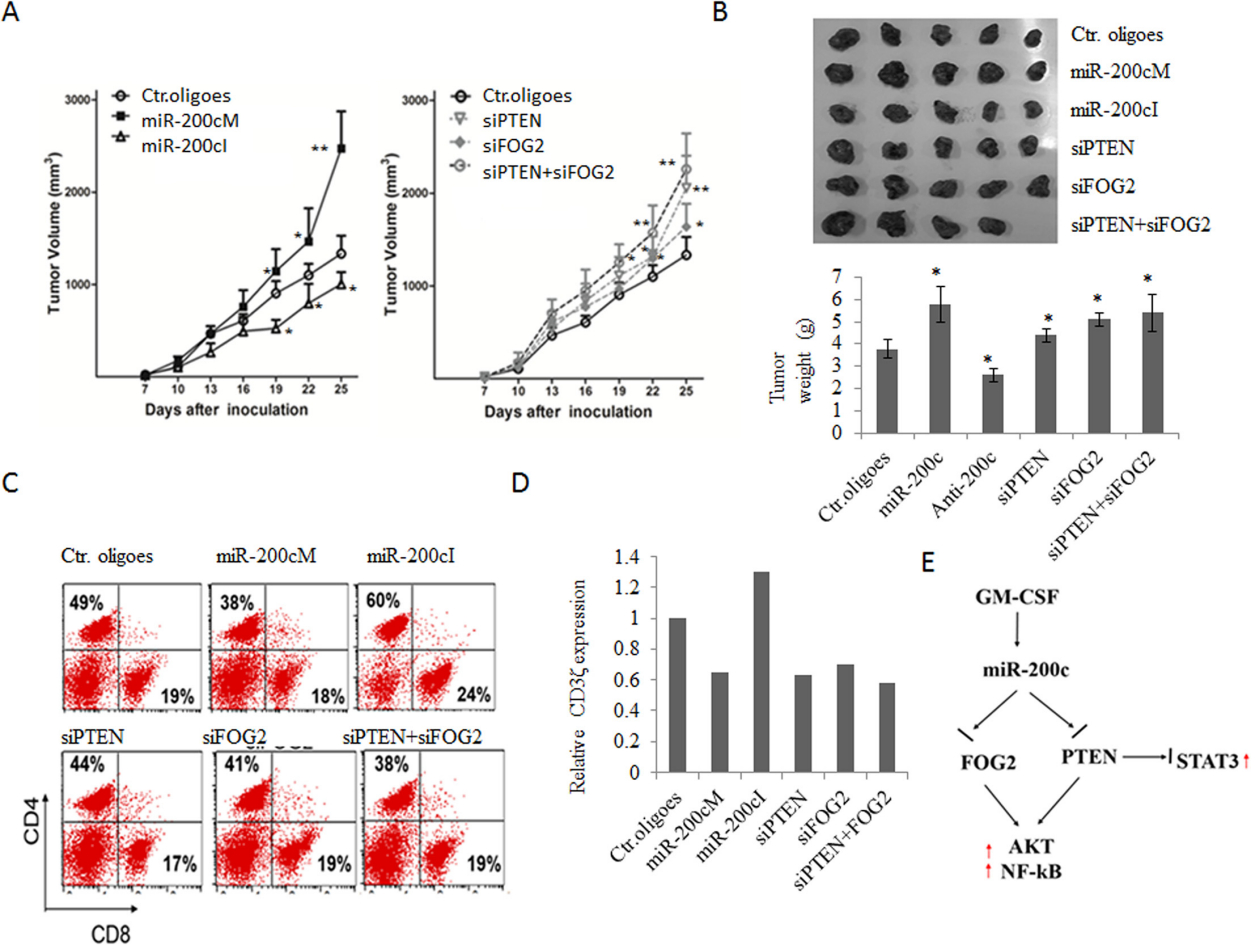
MiR-200c promotes tumor-induced immune tolerance. (A and B) Tumor growth curve (A), tumor size and tumor weight (B) in different groups. CT-26 tumor cells (1×10^6^) were subcutaneously injected into male BALB/c mice (*n* = 10). Then, 2 × 10^6^ pretreated MDSCs were intratumorally injected on days 5, 12 and 19 after tumor inoculation, and sacrificed on day 21. MDSCs were respectively pretreated with miR-200c mimics (miR-200cM), miR-200c inhibitors (miR-200cI), FOG2 siRNA (siFOG2), PTEN siRNA (siPTEN), siFOG2 and siPTEN (siFOG2+ siPTEN) or control oligoes (Ctr. oligoes). (C) The proportion of CD4^+^ and CD8^+^ T cells in inguinal lymph node of mice bearing tumor after injecting with differently treated MDSCs. (D) Expression of ζ-chain in T cells from the spleens of mice bearing tumor after injecting different-treated MDSC. Relative ζ-chain expression represents the percentages of geometric mean in experimental groups as compared to the geometric mean in control groups. (E) Proposed mechanism for the effects of miR-200c on the differentiation and suppressive function of MDSCs. In tumor environment, tumor released GM-CSF upregulates the expression of miR-200c. The upregulated miR-200c may target PTEN and FOG2 to promote the differentiation and suppressive function of MDSCs via inhibiting activation of STAT3 and activating Akt and NF-κB signals. Data shown here are representative results from at least three independent experiments. *, p<0.05, **, P<0.01.

Finally, we set up a mouse tumor challenging model to examine *in vivo* suppressive function of miR-200c transfected MDSCs. MDSCs were transfected with miR-200c mimics or inhibitors and then injected intratumorally on days 5, 12 and 19 after tumor inoculation into recipient mice. We found that tumors grew faster in mice infused with MDSCs transfected with miR-200c mimics than those injected with control oligoes transfected MDSCs; whereas tumor growth was significantly slower in mice injected with miR-200c inhibitors transfected ones (Fig 7A and 7B), suggesting that miR-200c promoted the immune suppressive function of MDSCs. Supporting above conclusion, the proportions of CD8^+^ T and CD4^+^ T cells in inguinal lymph nodes of mice injected with miR-200c mimics modified MDSCs were higher than those in mice with control MDSCs (Fig 7C). Since the downregulation of CD3ζ chain expression in T cells from the spleens of mice bearing tumor are often related to tumor immune suppression[33], we also evaluated the CD3ζ chain expression in T cells from the spleens of mice bearing CT-26 colon carcinoma. The levels of CD3ζ expression in CD4+ and CD8+ T cells was lower in mice treated with miR-200c mimics transfected MDSCs as compared to those in control group; whereas there was higher in the mice treated with miR-200c inhibitors transfected MDSCs (Fig 7D and Fig D in S1 File). Similar results could be found in mice bearing tumor after inoculation with PTEN or FOG2 siRNA transfected MDSCs (Fig 7D and Fig D in S1 File). Thus, miR-200c indeed improves the suppressive function and differentiation of MDSCs.

## Discussion

In this study, we demonstrate that miR-200c expressed in myeloid cells can regulate the suppressive function and differentiation of MDSCs by targeting PTEN and FOG2 expression. We have also found that myeloid cells associated miR-200c can be upregulated by tumor associated factor GM-CSF. These results may be used as a basis to improve anti-tumor immunotherapy.

MDSCs play a central role in tumor-mediated immune suppressive network during cancer progression. There is a dynamic interplay between lineage-specific miRNAs and intracellular signal pathways in the development and differentiation of myeloid lineage. The dysregulated miRNAs expression may result in an abnormal differentiation and accumulation of immature myeloid cells. Indeed, several miRNAs contribute to the expansion and suppressive function of MDSCs under pathophysiological conditions such as miR-155 [35], miR-21 [35], miR-142-3p [36], miR-494 [18, 22], miR-17-5p [22] and miR-20a [22]. Furthermore, these miRNAs also affect tumor growth by interrupting the differentiation and function of MDSCs [22]. We found that miR-200c also positively regulates MDSC differentiation and function. MiR-200c inhibitors may alleviate the immune suppressive potential of MDSCs to retard tumor growth. Thus, these miRNAs can serve as potential treatment targets of anti-tumor immune therapy.

MiRNAs in MDSCs mainly focus on PI3K/Akt and STAT3 signal pathways[1, 34]. STAT3 is a main transcriptional factor in the process of MDSC differentiation with the ability to drive CEBPβ, BCL-XL, c-myc, survivin and cyclin D1 expression [1, 34]. PI3K/Akt signaling pathway is also critical in the proliferation and survival of MDSCs through regulating the expression of transcription factors and genes [1, 34]. Notably, PI3K and STAT3 signal pathway may form a complex signaling network with mTOR/PTEN signals in regulating the differentiation and function of MDSC [37]. PTEN, a protein/lipid phosphatase, is the most important negative regulator of PI3K/Akt signaling pathway [25]. While FOG2 is found to bind to a regulatory subunit of PI3K, It is also acted a negative modulator of the PI3K/Akt pathway [34]. Studies show that some MDSC associated miRNAs could interrupt the activity of these signal pathways such as that miR-494 promotes the accumulation and function of tumor-expanded MDSC via targeting PTEN, which results in the subsequent activation of the Akt, NF-κB, and mTOR pathways. MiR-155 and miR-21 can promote STAT3 activation via targeting SHIP-1 and PTEN, respectively, synergistically leading to MDSC expansion [1, 34]. Our previous studies also show that miR-17-5p and miR-20a may alleviate MDSC suppressive potential by reducing STAT3 expression [22]. miR-200c is found to regulate the expression of PTEN and FOG2 to enhance the suppressive potential and promote the generation of MDSCs.

We found that GM-CSF is an effective inducer of miR-200c in tumor environment. GM-CSF is generally known as a cytokine capable of inducing the generation of granulocytes, monocytes and dendritic cells from BM precursors. However, it can also promote the formation of immune suppression within the tumor microenvironment. Under steady state, GM-CSF keeps at a low concentration in circulation and augments the systemic anti-tumor immune response. Meanwhile, during tumor initiation and progression, a high level of GM-CSF is necessary to drive the development of CD11b^+^ Gr-1^+^ cells that lead to immune suppression[38, 39]. Studies show that tumor-derived GM-CSF may be as one of the key factors in the generation of MDSCs both *in vivo* and *in vitro* [40, 41]. There is a direct correlation between secretion of GM-CSF in patients with head and neck cancers and presence of circulating immune suppressive GM progenitor cells [42]. Tumor cell-derived GM-CSF is also suggested to drive the accumulation of suppressive CD11b^+^ Gr-1^+^ myeloid cells in pancreatic cancers [30, 43]. Its blockade and knockdown inhibits the recruitment of CD11b^+^ Gr-1^+^ cells to the tumor microenvironment and arrest tumor development[30].

In conclusion, our data suggest a working model of miR-200c in regulating the activation of tumor-expanded MDSCs (Fig 7E). Tumor-derived GM-CSF induces the expression of miR-200c, which targets and negatively regulates FOG2 and PTEN expression. Down-regulation of FOG2 and PTEN promotes the activation of Akt and its downstream signaling pathways to enhance the expansion and immune suppressive potential of MDSCs.

## Supporting Information

S1 File A PTEN 3'-UTR sequence cloned into downsteam of firefly luciferase cassette in pSiCHECK-2 vector (Fig A). Generation of MDSCs (**Fig B**). PTEN (A) and FOG2 (B) mRNA 3’UTR may be bound by miR-200c (**Fig C**). Expression of ζ-chain in T cells from the spleens of mice bearing tumor after injecting different-treated MDSC (**Fig D**). Primers used in this study (**Table A**).
(DOCX)
