# Supplementary material for: MicroRNA-200c Promotes Suppressive Potential of Myeloid-Derived Suppressor Cells by Modulating PTEN and FOG2 Expression
Source: PLoS One. 2015 Aug 18;10(8):e0135867. doi: 10.1371/journal.pone.0135867 (PMC4540422; doi:10.1371/journal.pone.0135867)
Supplement: S1 File — Generation of MDSCs (Fig B). PTEN (A) and FOG2 (B) mRNA 3’UTR may be bound by miR-200c (Fig C). Expression of ζ-chain in T cells from the spleens of mice bearing tumor after injecting different-treated MDSC (Fig D). Primers used in this study (Table A). (DOCX) [file pone.0135867.s001.docx]

Supplementary Information

**MicroRNA-200c Promotes Suppressive Potential of Myeloid-derived Suppressor Cells by Modulating PTEN and FOG2 Expression**

Shiyue Mei^1^, Jiaxuan Xin^1^, Yu Liu^1^, Yuan Zhang^1^, Xue Liang^1^, Xiaomin Su^1^, Hui Yan^1^, Yugang Huang^1^ and Rongcun Yang^1,2,3*^

^1^Department of Immunology, Nankai University School of Medicine, Nankai University, Tianjin, P. R. China

^2^State Key Laboratory of Medicinal Chemical Biology, Nankai University, Tianjin, P. R. China;

^3^Key Laboratory of Bioactive Materials Ministry of Education, Nankai University, Tianjin, P. R. China.

*Corresponding author: Email: [ryang@nankai.edu.cn](mailto:ryang@nankai.edu.cn) (RY)

Funding: This research was supported by NSFC grants 31470876, 91029736, 91442111 and ISF-NSFC program 31461143010; a Ministry of Science and Technology grant (863 program, 2008AA02Z129); the National Key Scientific Program (2011CB964902) and the Program for Changjiang Scholars and Innovative Research Team in University (No. IRT13023) and State Key Laboratory of Medicinal Chemical Biology.

**Figure A. A PTEN 3' -UTR sequence cloned into downsteam of firefly luciferase cassette in pSiCHECK-2 vector.** The gated region was a targeting sequence by miR-200c.

**Figure B. Generation of MDSCs.** MDSCs were generated from BM cells by culturing with different tumor-derived factor combinations (GM-CSF alone, GM-CSF with IL-6, TNFa, or IL-4 plus PGE2). After four days, the proportion of Gr-1^+^CD11b^+^ cells was analyzed by FAScan. The figures shown here were at least representative results from three independent experiments.

**Figure C. PTEN (A) and FOG2 (B) mRNA 3’UTR may be bound by miR-200c.** This direct pairing was validated and reported before [1].

**
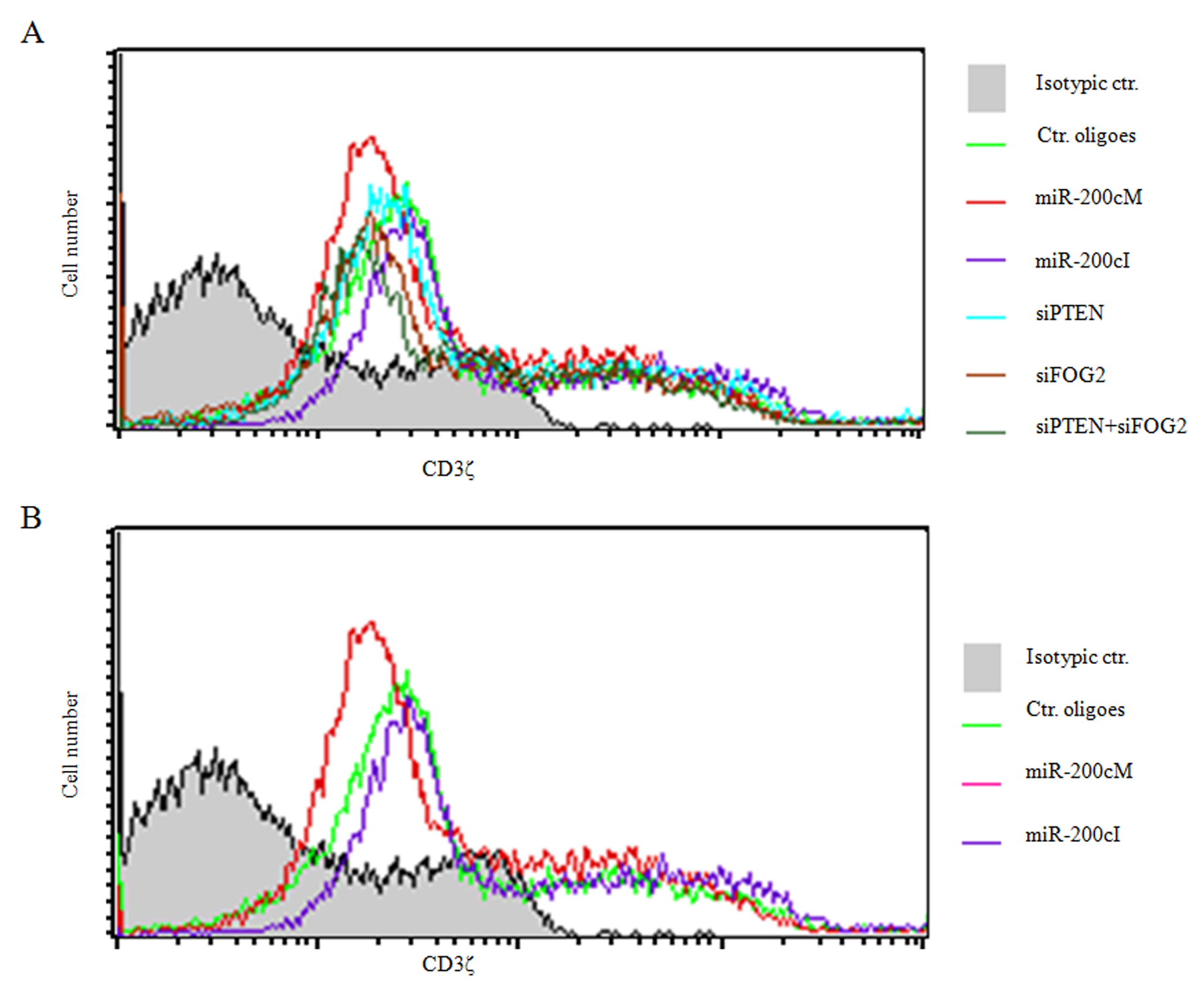
**

**Figure D. Expression of ζ-chain in T cells from the spleens of mice bearing tumor after injecting different-treated MDSC.** (A and B) Expression of ζ-chain in T cells from the spleens of mice bearing tumor after injecting control oligoes (ctr.oligoes), miR-200c mimics (miR-200cM), miR-200c inhibitor (miR-200cI), PTEN siRNA (siPTEN), FOG2 siRNA (siFOG2) or PTEN siRNA and FOG2siRNA -treated MDSC. T cell surface marker (CD4 and CD8) staining was performed prior to fixation. The fixed and permeabilized cells were stained using anti- CD3- ζ mAb. Expression of ζ-chain in CD4+ and CD8+ T cells from the spleens of mice was analyzed using FAScan. Isotypic ctr., isotypic control.

**Table A. Primers used in this study.**

| Primer | Sequence (5'to3') |
| --- | --- |
| miR-200c stem-loop primer | GTCGTATCCAGTGCAGGGTCCGAGGTATTCGCACTGGATACGAC TCCATC |
| miR-200c qPCR Forward primer | AGTGACGTAATACTGCCGGGT |
| Universal qPCR Reverse primer | CCAGTGCAGGGTCCGAGGTA |
| U6 qPCR Forward primer | CTCGCTTCGGCAGCACA |
| U6 qPCRReverse primer | AACGCTTCACGAATTTGCGT |
| pri-miR-200c qPCR Forward primer | TGATCTTGAAGGTGGACTGG |
| pri-miR-200c qPCR Reverse primer | CACTGGATTGGAGGAGGG |
| S100A8 qPCR Forward primer | CCGTCTTCAAGACATCGTTTGA |
| S100A8 qPCR Reverse primer | GTAGAGGGCATGGTGATTTCCT |
| mus S100A9 qPCR Forward primer | TCATCGACACCTTCCATCAATACTC |
| mus S100A9 qPCR Reverse primer | GAGGGCTTCATTTCTCTTCTCTTTC |
| Arg1 qPCR Forward primer | TCCACCCTGACCTATGTGTCATTT |
| Arg1 qPCRReverse primer | CGTCTCGCAAGCCAATGTACA |
| iNOSqPCR Forward primer | CACCTTGGAGTTCACCCAGT |
| iNOSqPCR Reverse primer | ACCACTCGTACTTGGGATGC |
| STAT3 qPCR Forward primer | GAGAACCTCCAGGACGACTTTGAT |
| STAT3 qPCR Reverse primer | TCTTAATTTGTTGGCGGGTCTGA |
| PTEN qPCR Forward primer | GTCCAGAGCCATTTCCATCCT |
| PTEN qPCR Reverse primer | TCTTTCTGCAGGAAATCCCAT |
| Fog2 qPCR Forward primer | GAGCTGCGAAGACGTGGAGT |
| Fog2 qPCR Reverse primer | CCAGGCTGTCCTGGTTTGTC |
| GAPDH qPCR Forward primer | GGTGAAGGTCGGTGTGAACG |
| GAPDH qPCR Reverse primer | CTCGCTCCTGGAAGATGGTG |
| PTEN 3-UTR Forward primer | ATACCGCTCGAGTGAAGGTCTGAATGAGGGTT |
| PTEN 3-UTR Reverse primer | AAGGAAAAAAGCGGCCGCGTGCCACAGCAAAGAATG |
| PTEN Mut 3-UTR Forward primer | TCTTTATGACTCATAACACGATTAGCCTGAAGTGC |
| PTEN Mut 3-UTR Reverse primer | GCTAATCGTGTTATGAGTCATAAAGAAAGTTTGAA |

**Supplementary References**

1. Park, J. T., M. Kato, H. Yuan, N. Castro, L. Lanting, M. Wangand, and R. Natarajan. 2013. FOG2 Protein Down-regulation by Transforming Growth Factor-β1-induced MicroRNA-200b/c Leads to Akt Kinase Activation and Glomerular Mesangial Hypertrophy Related to Diabetic Nephropathy. Journal of Biological Chemistry 288: 22469-22480.
